# Severe *Staphylococcus aureus* infection: associated factors and outcomes

**DOI:** 10.1016/j.bjid.2025.104573

**Published:** 2025-08-09

**Authors:** Narendra Babu Valobdás, Marcelo Ribeiro Alves, Erica Aparecida dos Santos Ribeiro da Silva, Maria Cristina da Silva Lourenço, Beatriz Coelho de Negreiros Nascimento, Valdilea Gonçalves Veloso, Sandra Wagner Cardoso, Cardoso, Cristiane da Cruz Lamas

**Affiliations:** a Instituto Nacional de Infectologia Evandro Chagas, Fiocruz, Rio de Janeiro, RJ, Brazil; b Instituto Nacional de Cardiologia, Rio de Janeiro, RJ, Brazil; c Universidade do Grande Rio (UNIGRANRIO), Rio de Janeiro, RJ, Brazil

**Keywords:** *Staphylococcus aureus*, HIV/AIDS, MRSA, Bacteremia, Mortality

## Abstract

**Introduction:**

*Staphylococcus aureus* causes potentially life-threatening infections, with a somber prognosis when the infection is caused by methicillin-resistant *S. aureus* due to limited treatment options. The present study describes serious infections by *S. aureus* in patients hospitalized in an infectious disease’s unit in Rio de Janeiro, Brazil, between 2016 and 2021.

**Material and methods:**

This was a retrospective study based on data from positive samples diagnosed by the microbiology laboratory and by review of medical records. Clinical-demographic variables and outcomes were compared between Patients Living with HIV (PLHIV) and non-HIV patients. Data were analyzed using Jamovi 1.6 and R 4.0.1 statistical software.

**Results:**

A total of 67 patients with a serious *S. aureus* infection were identified, of whom 29 presented bacteremia and 38 other infections. Thirty-one of 67 (46.3%) were PLHIV. The median age of all patients was 46years, although PLHIV were significantly younger than non-HIV individuals (36 vs. 60 years-old, *p* < 0.001). The median CD4 lymphocyte count was 95 cells/mm^3^. Community infection occurred in 36/67 (53.7%) patients, of whom 19/36 (52.7%) had bacteremia. A total of 20 MRSA infections (29.9% of the patients) were identified, which accounted for 14/36 (38.8%) of the community infections. More than a third of PLHIV (38.7%) had MRSA, and all these were sensitive to cotrimoxazole. No difference in mortality was found between PLHIV and non-HIV patients, nor between the MRSA and MSSA groups. Bacteremia was present in 29 patients; MRSA accounted for 9 (31.0%) of these. The 30-day mortality was 4/9 (44.4%) and 2/20 (10%) in MRSA and MSSA bacteremia, respectively.

**Conclusions:**

The most frequent comorbidity in patients with severe *S. aureus* infections was HIV, with a high rate of MRSA infections recorded in PLHIV. PLHIV were younger, but did not suffer higher mortality, although they did have more relapses and new staphylococcal infections.

## Introduction

*Staphylococcus aureus* is a bacterium that is widely recognized as one of the main causes of infections of the skin and soft tissues that affect patients in the community and in healthcare associated scenarios. .^1^ Infection by *S. aureus* is potentially life-threatening. The bacterium is frequently found in the skin and mucous membranes, colonizing 15 %–36 % of the general population,^2^ with persistent colonization in about 10 %. The relationship between colonization and infection is confirmed by the fact that strains of *S. aureus* isolated from the nose and those identified in infections in the same individual are usually genetically identical, and can thus be considered to be endogenous.^3^ The groups most affected by this infection include those with the Acquired Immunodeficiency Syndrome (HIV/AIDS), Chronic Renal Disease (CRD) that requires Hemodialysis (HD), and illicit drug users.^4^ People Living with HIV/AIDS (PLHA) have an increased risk of colonization and/or infection by MRSA, most frequently affecting skin and soft tissues. The risk factors in this population include advanced immunosuppression (CD4 count of <50 cells/mm^3^), high plasma viral load (>100,000 copies per milliliter), and an absence of antiretroviral therapy.^5^^,^^6^

Methicillin-Resistant *Staphylococcus Aureus* (MRSA) has an auxiliary Penicillin-Binding Protein – PBP2 or PBP2C, codified by the MECA and MECC genes, respectively – which has reduced affinity for beta-lactam agents. Resistance is usually diagnosed using procedures recommended by the European Committee on Antimicrobial Susceptibility Testing[7] or its Brazilian counterpart, BRCAST. This diagnosis can be phenotypic, based on the Minimum Inhibitory Concentration (MIC) or disk-diffusion test, a routine laboratory method which may use latex agglutination to detect the presence of PBP2a more reliably than PBP2c. Genetic detection by PCR is also considered to be a reliable approach.^7^ Infections by MRSA are a global public health problem and are distinct from infection by MSSA (Methicillin-Sensitive *S. aureus*) due to the limited treatment resources available, leading to greater demands on public health systems and worse clinical outcomes.^8^, ^9^, ^10^ The presence of MRSA in the community (community-acquired MRSA, or ca-MRSA) has increased significantly in recent decades, a tendency also observed in PLHIV, who show greater ca-MRSA colonization and infection rates than the general population.^11^^,^^12^

The present study describes the *S. aureus* infection profile of patients hospitalized in the Instituto Nacional de Infectologia Evandro Chagas, Rio de Janeiro, between 2016 and 2021. This study is important because of the relative paucity of epidemiological data on the infection profile of *S. aureus* in Brazil, in both the general population and, particularly, in PLHIV.

## Methods

### Type and place of study

This study was based on a retrospective analysis of patients in Instituto Nacional de Infectologia Evandro Chagas, Rio de Janeiro, Brazil. This institution, which specializes in clinical research, teaching, and reference services in infectious diseases, is a national reference center for the care of patients with HIV/AIDS, with a cohort of approximately 4000 PLHA monitored since the 1980s.

### Inclusion criteria

The present study focused on adult patients hospitalized in the infirmary (22 beds) or in the Intensive Care Unit (ICU) (four beds). Patients were selected based on the following criteria – (i) At least 18-years of age, (ii) In need of hospitalization for more than 48 hours, and (iii) Diagnosis of *S. aureus* infection between January 2016 and December 2021.

### Data collection

A standard case report form was prepared (Supplementary Material) for data collection. All the patients admitted for treatment were screened for colonization by *S. aureus* using the phenotype for diagnostic identification and sensibility testing.

The patients were identified through the microbiological data on both sterile (blood, articular liquid, pleural liquid, pericardial fluid and cerebrospinal fluid) and non-sterile samples (tracheal aspirate and bronchoalveolar lavage, and secretions of cutaneous abscesses), extracted from the reports of the Laboratory of Bacteriology and Bioassays. The sample results were ranked in the following order of priority: blood, respiratory, skin, and soft tissue samples. Following this identification, information on the clinical-demographic profile of the patients was searched for. Only patients considered to have a serious or clinically relevant *S. aureus* infection were included, defined as an infection that had systemic repercussions with the need for hospitalization and intravenous antibiotic treatment, whether associated or not with bacteremia.

In the case of patients with more than one infection site, consideration was given to reporting sensitivity to the infection site with the poorest prognosis and greatest clinical significance.

### Definitions

- Index event: *S. aureus* infection.
- Clinically relevant *S. aureus* infection or serious infection was defined as that which had systemic repercussions requiring hospitalization and intravenous antibiotic therapy, with or without associated documented bacteremia.
- Hospital-acquired infection: infection occurring after 72 hours of hospitalization, respecting the incubation period of diseases.^13^
- Community infection: infection present at the time of hospitalization or identified within the first 72 hours of hospitalization.^13^
- Recurrence or relapse of bacteremia was defined as infection manifested within 30-days of the first episode, after negative blood cultures, and reinfection as bacteremia 30-days after the first episode.^14^
- Death within 30-days was defined as the time interval between *S. aureus* infection and death.

### Statistical analysis

The data were expressed as frequencies and percentages (for categorical and nominal variables), or the median and interquartile interval (for continuous variables). The categorical variables were analyzed using the Chi-Square test, while the continuous variables were evaluated using Mann-Whitney’s *U*. A significance of 5 % was considered for all analyses. These analyses were employed to identify factors associated with the different outcomes related to the study variables, i.e., sex, age, CD4 count, viral load, associated comorbidities, alcoholism, malnutrition, obesity, focus of the infection, and the sensitivity profile to antimicrobial drugs. All data were analyzed with the Jamovi 1.6 and R 4.0.1 statistical software. Ethical approval was given by INI Evandro Chagas Ethics Committe under CAAE number 52,792,321.5.0000.5262, on 25 October 2021.

## Results

A total of 193 patients with positive *S. aureus* cultures were identified in the study period. However, 126 patients were excluded from the study, including 14 that were not registered in the institution’s electronic records system and 112 who were not hospitalized, as they were seen in the emergency service department or in the outpatient units.

**Fig. 1:**
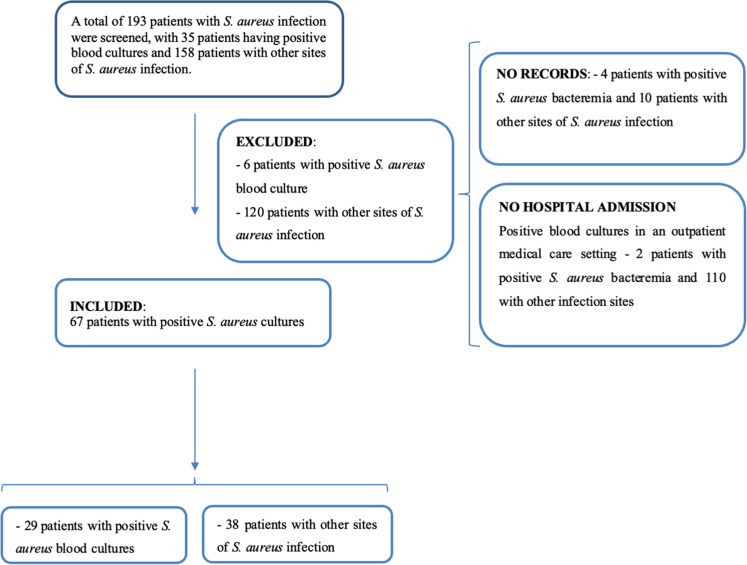
Flow chart of the study selection and inclusion process of patients infected with *S. aureus* based on culture results.

After eliminating the patients that did not satisfy the selection criteria, a total of 67 cases of serious *S. aureus* infection were consolidated for the present study (Fig. 1). Overall, 29 of these patients had positive blood cultures, while the other 38 presented other infections sites, such as cutaneous lesions or abscesses, bronchoalveolar lavage and/or tracheal secretion, ophthalmic secretions, articular fluid, bone fragments, and urine.

Overall, 31 of the 67 patients (46.3 %) were PLHIV. Two cases of relapse (6.5 % of the total) and three new infections (9.7 %) were identified, with all five cases recorded in PLHA. Just over half the sample or 37 of the 67 patients (55.2 %) were men, and 30 (44.8 %) were women, while 69.4 % declared that their ethnicity was black. Similar proportions were observed in the PLHIV and non-HIV groups.

The median age of the patients was 46-years (Inter-Quartile Range [IQR = 31–62 years]), although the PLHIV group was significantly younger when compared to the non-HIV individuals (36-years [IQR = 26–47.5 years] vs. 60-years [IQR = 43–66.25 years], *p* < 0.001).

The vast majority (79.1 %) of the patients in both groups lived in the city of Rio de Janeiro, with a median of three persons (IQR = 2–4) living in the same residence. About half, i.e., 34 of the 67 patients (50.7 %) with *S. aureus* infection had graduated primary school, while less than a third (20 or 29.8 %) were high school graduates. Only 15 patients (22.3 %) were formally employed and 17 (25.3 %) declared having a family income of less than one minimum wage at the time of hospitalization, reflecting the socioeconomic vulnerability of many of these patients.

### Infection by MRSA and MSSA

Ca-MRSA accounted for 20 (29.9 %) of the 67 patients with *S. aureus* infection, considering both the hospital and community cases. The definition of ca-MRSA was based on phenotypic antibiotic sensitivity, with all (100 %) being sensitive to cotrimoxazole and doxycycline, and 90 % to clindamycin. Of the 47 patients with MSSA, 97.9 % showed sensitivity to cotrimoxazole, 95 % to doxycycline, and 55.3 % to clindamycin.

Patients with MRSA infections received antibiotics intravenously for a median period of 5-days (IQR = 3.25–8 days) and orally for 7days (IQR = 5.25–9.5 days), whereas those with MSSA infection received antibiotics intravenously for 6days (median, IQR = 3–14 days) and orally for 7days (IQR = 6.75–11.75 days). The most frequent antibiotics administered intravenously to treat MRSA infection were (in decreasing order of frequency) vancomycin in 11 of the 20 patients (55 %), clindamycin in 5 (20 %), and daptomycin, linezolid, and co-trimoxazole, which were given to one patient each (5 %). None of these patients were given oxacillin or any other beta-lactam antibiotics. In the patients with MSSA infection, the following antibiotics were given intravenously (in decreasing order of frequency) – oxacillin in 12 of the 47 patients (25.5 %), vancomycin in 9 (19.1 %), cefazolin in 3 (6.4 %), and cotrimoxazole in two (4.3 %); amoxicillin with clavulanic acid, clindamycin, daptomycin, and teicoplanin were each given to one patient each (2.1 %).

Of the 67 patients included in the study, eight cases of *S. aureus* abscess in soft tissues were identified. Half of the abscesses were caused by MRSA, while the other half were caused by MSSA. Seven of the eight abscesses underwent bedside drainage.

The median length of hospital stay for patients with MRSA was 18days (IQR 12‒21.5), while for those with MSSA it was 25 days (IQR 13.5‒38.5, p-value = 0.134).

There was no statistical difference in intensive care outcomes and 30-day mortality between patients with MRSA and MSSA infections. Some patients with MRSA infection required respiratory support with mechanical ventilation (30 %), needed non-invasive ventilation (20 %), and 30 % required treatment with amines, with similar proportions of these treatments given to patients infected with MSSA (Table 1). Although the 30-day mortality rate was higher (30 %) in the MRSA group compared to the MSSA group (19.6 %), the difference was not statistically significant (*p* = 0.542).

**Table 1:** Selected outcomes and 30-day mortality of patients with *Staphylococcus aureus* infection, stratified as MRSA and MSSA.

	Number (%) of patients infected with		
Clinical parameter	MRSA	MSSA	p
ICU admission	7 (35.0 %)	21 (44.7 %)	0.642
Use of amines (vasopressors)	6 (30.0 %)	12 (29.8 %)	1
Mechanical ventilation	6 (30.0 %)	14 (30.4 %)	1
Non-invasive ventilation	4 (20.0 %)	8 (19.0 %)	1
Acute renal failure in ICU (requiring hemodialysis)	4 (20.0 %)	13 (28.3 %)	0.690
Median (IQR) time in ICU (in days)	11 (9)	11 (9)	0.852
Death at 30 days	6 (30.0 %)	9 (19.1 %)	0.542

MRSA, Methicillin-Resistant *Staphylococcus Aureus*; MSSA, Methicillin-Sensitive *Staphylococcus Aureus*; ICU, Intensive Care Unit; IQR, Interquartile Range.

### Portals of entry, infection sites, and bacteremia

The main portals of entry for *S. aureus* infections were chronic dermatological conditions, such as eczema, psoriasis, and prurigo, which were identified in 20 cases (30 %). Short-term central intravenous catheters were the portal of entry in eight cases (11.9 %), showing local signs of inflammation. Other presumed portals of entry were hemodialysis central catheters (6 %), bedsores (4.5 %), peripheral vascular catheters (4.5 %), and traumas, burns, and surgery, accounting for one case each (1.5 %).

Short term central venous catheters were the main portal of entry for bacteremia in eight of the 29 cases (27.6 %), in comparison with zero cases in patients with other sites of infection (*p* = 0.002). By contrast, chronic dermatological conditions were identified as the most common source of infection in patients with other sites of infection, with 13 cases (34.2 %) among the 38 patients, in comparison with 7 (24.1 %, *p* = 0.533) in the 29 patients with staphylococcal bacteremia*.*

Overall, 29 (43.3 %) of the patients diagnosed with *S. aureus* infection developed bacteremia. However, no difference in complications and mortality was found when comparing patients with bacteremia and those with other sites of infection and no bacteremia. For example, similar proportions of patients with (44.8 %) and without bacteremia (39.4 %) required intensive care. Similar rates were also found in the two groups regarding the use of vasopressor amines, mechanical and non-invasive ventilation, acute renal failure and the need for hemodialysis. The 30-day mortality rate was 20.6 % in patients with bacteremia and 23.6 %in those with *S. aureus* infections in other sites, with no significant difference between the two groups. However, significant differences were found when the cases of bacteremia and other occurrences were considered in the context of infection by MRSA or MSSA. Nine of the 29 (31 %) patients with bacteremia, presented resistance to oxacillin indicating MRSA infection, and 4/9 (44.4 %) died within 30-days. In the other 20 patients with MSSA bacteremia, however, only 2 (10 %) died. Overall, 17 (58 %) of the 29 patients with bacteremia had an echocardiogram performed (either transesophageal or transthoracic, as this was not specified in the medical records), while 15 (51 %) had control blood cultures collected.

### HIV/AIDS and other comorbidities

Thirty one of 67 patients (46.3 %) with *S. aureus* infection were PLHIV. The median CD4 count was 95 cells/mm^3^ (IQR = 40–616), while the median viral load was 102 copies/mm^3^ (IQR = 39‒12,180.5). Most (80.6 %, 25/31) of the PLHIV were on Antiretroviral Therapy (ARVT). Seven of 31 (22 %) PLHIV were colonized with MRSA when admitted to hospital, while 20 (64.5 %) were infected with *S. aureus* soon after admission, while the other 11 (35.4 %) were infected more than 72 hours after admission.

Staphylococcal infections in PLHIV predominantly affected the skin and soft tissues, which accounted for 15/31 (48.4 %) of the patients, followed by bacteremia in 12 (38.7 %) patients and pneumonia in 8 (25.8 %). Similar proportions were recorded in the non-HIV patients, with infections of the skin and soft tissues in 12 of the 36 patients (33.3 %), bacteremia in 17 (47.2 %), and pneumonia in 8 (22.2 %).

MRSA infections were present in 38.7 % of PLHIV, and all of them (100 %) were susceptible to cotrimoxazole, doxycycline and linezolid, and 90 % to clindamycin, a phenotypic pattern typical of CA-MRSA. However, no information on patients who were on cotrimoxazole prophylaxis was evaluated, and a possible relationship between this prophylaxis and the susceptibility pattern of the bacteria in these patients was not further investigated.

Both PLHIV and non-HIV patients infected with *S. aureus* presented a range of comorbidities (Table 2). The most frequent comorbidities in both groups were diabetes mellitus, hypertension, and cancer, with similar proportions recorded in both groups. No significant differences in outcomes and mortality were recorded between the PLHIV and non-HIV patients. While 32.3 % of the PLHIV patients had intensive care, half (50 %) of the non-HIV patients were admitted to the ICU (*p* = 0.22). In addition, approximately one third of the patients of each group – both PLHIV and non-HIV – needed artificial ventilation with amines or hemodialysis. Similarly, the 30-day mortality rate in the PLHIV group was 25.8 %, while it was 20.0 % in the non-HIV group (*p* = 0.789).

**Table 2:** Comorbidities in hospitalized patients with *S. aureus* infection stratified by HIV status.

Variable^a^		Number (%) of patients		
	Total (*n* = 67)	non-HIV	PLHIV	p-value
Diabetes mellitus	12 (17.9 %)	7 (19.4 %)	5 (16.1 %)	0.973
Systemic arterial hypertension	9 (13.4 %)	7 (19.4 %)	2 (6.5 %)	0.232
Chronic renal disease	8 (11.9 %)	3 (8.3 %)	5 (16.1 %)	0.546
Congestive heart failure	5 (7.5 %)	4 (11.1 %)	1 (3.2 %)	0.448
Chronic hepatopathy	2 (3.0 %)	1 (2.8 %)	1 (3.2 %)	1.000
Coronary artery disease	1 (1.5 %)	1 (2.8 %)	0 (0.0 %)	1.000
Chemotherapy	3 (4.5 %)	1 (2.8 %)	2 (6.5 %)	0.894
Radiotherapy	5 (7.6 %)	4 (11.4 %)	1 (3.2 %)	0.429
Steroid use	6 (9.0 %)	4 (11.1 %)	2 (6.5 %)	0.813
Recent cancer	5 (7.5 %)	2 (5.6 %)	3 (9.7 %)	0.862
Cancer treatment	8 (11.9 %)	5 (13.9 %)	3 (9.7 %)	0.879
Hepatitis C	1 (1.5 %)	0 (0.0 %)	1 (3.2 %)	0.94
HTLV	6 (9.0 %)	5 (13.9 %)	1 (3.2 %)	0.273
Chronic dermatopathy^a^	6 (9.0 %)	5 (13.9 %)	1 (3.2 %)	0.273
COPD	1 (1.5 %)	1 (2.8 %)	0 (0.0 %)	1.000
Other^b^	19 (28.4 %)	11 (30.6 %)	8 25.8 %)	0.874

a PLHIV, person living with HIV, Chronic dermatopathy was defined as persistent cutaneous lesions for more than six weeks which were present at the time of the *S. aureus* infection/hospitalization (for example, psoriasis, chronic dermatitis, eczema, prurigo, and granulomatous skin diseases), COPD, Chronic Obstructive Pulmonary Disease.

b Other comorbidities include alopecia, glaucoma, transversal myelitis, Guillain-Barré syndrome, hernia and gastritis.

### Mortality and associated factors

**Fig. 2:**
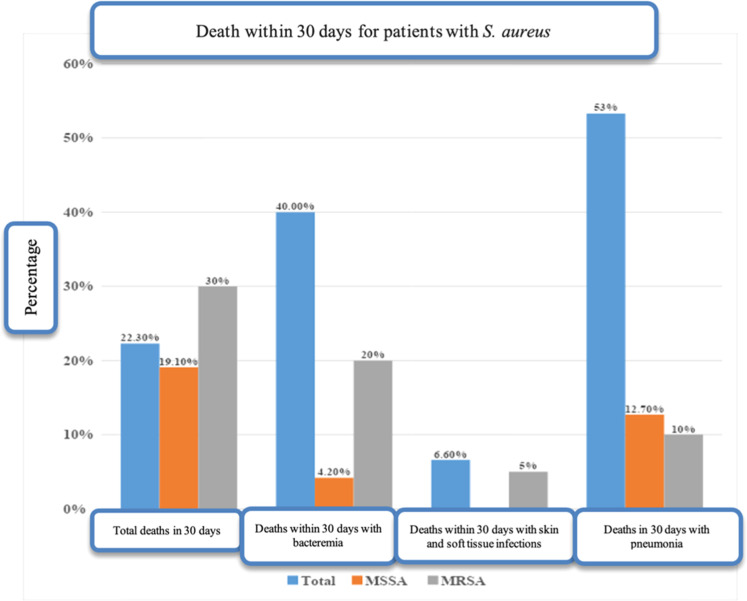
30-day mortality in patients with *S. aureus* infection, stratified by MSSA and MRSA infections, and disease presentation. MRSA, Methicillin-Resistant *Staphylococcus aureus*; MSSA, Methicillin-Sensitive *S. Aureus*; VAP, Ventilator Associated Pneumonia.

Overall, 15 of the 67 study patients (22.3 %) died within 30-days, of whom 8/15 (53.3 %) were patients with pneumonia on mechanical ventilation, while 6 (40.0 %) had bacteremia, and 1 (6.7 %) had a cutaneous lesion. Twelve of the 15 (80 %) deaths took place in the index hospitalization (Fig. 2).

Mortality associated risk factors are presented in Supplementary Table 1. A total of 15 of the 67 patients (22.3 %) died within 30-days. While there was a clear tendency for mortality in the older patients, with a median age of 58years (IQR = 32.5–63.0 years), in comparison with the patients that did not die (median age 44years, IQR = 32.5–61.5 years), the difference was not significant (*p* = 0.354). Mortality was similar in PLHIV (53.0 %) and in non-HIV patients (45.1 %), with no statistical difference (*p* = 0.78).

Some comorbidities were associated with mortality (Supplementary Table 2), but no statistically significant association was observed between any comorbidity and the outcome death in 30-day. No significant differences in 30-day mortality were found for any of the clinical or laboratory data (Supplementary Table 3). Most of the patients had stable vital signs (afebrile, normotensive, and eupneic) at hospital admission to the ward, probably because these patients were stabilized clinically in the emergency room before hospitalization. No statistically differences were found in C-Reactive Protein (CRP) and creatinine values at admission when comparing patients with a fatal and non-fatal outcome. The median CRP at admission was 10.84 mg/L in those who died, and 14.1 mg/L in those who survived (*p* = 0.592). The median creatinine concentration at admission was 1.09 mg/dL in the patients with a fatal outcome and 1.22 mg/dL in those who did not die (*p* = 0.488).

## Discussion

The present study describes the characteristics of patients with a serious *S. aureus* infection that required hospitalization in a Brazilian reference institution for infectious diseases. Based on laboratory records, the analyses focused on 67 patients who were admitted to hospital between 2016 and 2021. Almost half (46.3 %) of these patients were PLHIV. As this group has been the main population cared for in this service since the 1980s, their predominance in the sample analyzed here implies sampling bias. *S. aureus* infection is associated with a higher morbidity and mortality, in particular in PLHIV, who are more frequently hospitalized due to their vulnerability to opportunistic diseases, and are thus are also more frequently exposed to antibiotics, with associated immunosuppression. Nevertheless, few studies in Brazil or elsewhere have focused on this relationship, and we believe our study contributes to a better understanding of *S. aureus* infections in PLHIV.

### Sociodemographic features

Overall, the PLHIV and non-HIV groups had broadly similar sociodemographic characteristics, with a predominance of single males of black ethnicity. The majority only had primary school, while only a minority had high school diplomas or college degrees. More than two-thirds of the patients had a family income of less than three minimum wages, and 27.4 % had an income of less than one minimum wage, reflecting a disease profile of individuals in a scenario of social vulnerability. These findings were in agreement with those of a population-based study done by the Center for Disease Control and Prevention (CDC) in three major North American states showing that males of ethnic minorities and underprivileged socioeconomic status had the highest rates of *S. aureus* infection, in particular CA-MRSA.^15^

### Community vs. hospital acquired infections

Just over half of the patients involved in the present study (53.7 %) were infected by *S. aureus* at the time of their admission to the hospital (i.e., they had community-acquired infection). Regarding bacteremia, in 19/29 (65.5 %) infection was community-acquired, highlighting the relative frequency of hospital-acquired infections and the importance of infection control in this environment. In nine of the 29 cases of bacteremia, the condition was caused by MRSA, and in 6 (66.6 %) cases, infection was community-acquired, which is consistent with the findings of a Canadian study.^16^ This prospective study of 299 adult cases of *S. aureus* bacteremia between 2011 and 2013 found a prevalence of 66.9 % of infections caused by CA-MRSA.

### Bacteremia, echocardiography and control blood cultures

One frequent question concerning patients with *S. aureus* bacteremia is whether these cases are associated with endocarditis since endocarditis occurs in 6 %–32 % of these cases.^17^ In our study, only two cases of endocarditis were documented in patients with *S. aureus* bacteremia. However, only 17 of the 29 patients with bacteremia had an echocardiogram performed; the low frequency of echocardiography may be explained by the fact that patients were managed by infectious diseases specialists who judged it was not necessary due to a favorable clinical course and known portals of entry, as in the case of skin and soft tissue infections or those associated with vascular catheters. Moreover, no patients in the sample had valvular prosthetics or intracardiac devices, which would result in echocardiography being highly recommended. Importantly, guidelines recommend transthoracic echocardiogram for all cases of *S. aureus* bacteremia, whereas transesophageal echocardiogram would be recommended for patients with a high probability of endocarditis.^18^

Another critical aspect of *S. aureus* bacteremia is to establish clearance, that is, to determine blood culture negativity from samples obtained 72‒96 hours after the beginning of appropriate antibiotic treatment. Evidence of a lack of growth in these control blood cultures makes IE less probable. Only 15 (51 %) of patients with *S. aureus* bacteremia in our study had control blood cultures collected. Central venous catheters were the main portal of entry for bacteremia, recorded in 8 (27.6 %) cases but in none of the other 38 patients with other infection sites (*p* = 0.002). The presence of central venous catheters was identified as an independent risk factor for *S. aureus* bacteremia, as reported in multi-center studies conducted in tertiary hospitals in the United States, Ireland, and Canada, where between 56 % and 83 % of the cases of *S. aureus* bacteremia were associated with central venous catheters.^18^, ^19^, ^20^, ^21^, ^22^

Chronic dermatological conditions, with loss of cutaneous integrity, were present in 34.2 % of the cases of *S. aureus* infection at other sites. This is consistent with the available data, which show that a higher rate of community infection by *S. aureus* is found in patients with atopic or infectious dermatitis at the time of diagnosis of the staphylococcal infection, in particular conditions such as cellulitis and abscesses.^23^^,^^24^

The most common comorbidities seen in patients with a clinically relevant infection by *S. aureus* in the present study were HIV/AIDS, diabetes mellitus, chronic renal disease, and recent cancer. This is in line with the published literature, where risk factors for colonization or infection by *S. aureus* include diabetes mellitus (associated with reduced immunity and/or the use of subcutaneous insulin), acquired immunodeficiency (such as PLHIV), the presence of chronic cutaneous lesions, chronic renal disease that requires hemodialysis, and the use of intravenous drugs.^25^^,^^26^

### Comparison of PLHIV and non-HIV positive individuals

PLHIV included in the present study were significantly younger than the non-HIV patients, with a median of 36 vs. 60 years (*p* < 0.001), which may indicate that the risk of a serious *S. aureus* infection is more precocious in the PLHIV population. Overall, 22 % of the PLHIV with a clinically relevant *S. aureus* infection were colonized by MRSA at admission, a rate higher than that reported in a systematic review on MRSA colonization in PLHIV the region of the Americas, of 10 %.^27^

*S. aureus* bacteremia was seen in 12 of the 31 PLHIV patients (38.7 %), a proportion similar to that observed in non-HIV patients (17/36, 47.2 %). Some studies have shown that PLHIV have a significantly higher frequency of bacteremia than non-HIV individuals. Larsen et al. (2012a),^28^ for example, reported an incidence of bacteremia in patients infected with HIV of 494 cases per 100,000 individuals per year. This study monitored a cohort of 97,000 adult Danes between 1995 and 2007, including individuals both infected with HIV and not infected, and found that, even in the absence of injectable drugs, the risk of bacteremia in the HIV positive patients was 24 times higher than that of the non-HIV population. Although the use of injectable drugs contributes to this risk, no reports of the use of these drugs were recorded in the present study. A CD4 cell count of less than 100 cells/mm^3^ was also identified as a strong independent risk factor for *S. aureus* bacteremia for PLHIV,^5^ consistent with the findings of the present study which showed a median CD4 count of 95 cells/mm^3^.

### MRSA colonization and infection

In our study, MRSA accounted for 20 of the 67 staphylococcal infections (29.9 %). Fourteen of these 20 patients (70 %) had ca-MRSA infection. All MRSA were susceptible to cotrimoxazole and doxycycline, and 90 % to clindamycin, which indicates that the MRSA had a phenotype consistent with that of ca-MRSA, even when the infection was acquired in the hospital environment. Noteworthily, all 47 MSSA isolates presented a susceptibility of 97.9 % for cotrimoxazole and 95 % for doxycycline, supporting the use of these drugs for the transition to oral treatment of serious staphylococcal infections.

At the time of hospital admission, 14 of the 20 patients (70 %) infected with MRSA were colonized by this pathogen. Individuals with MRSA colonization act as reservoirs for transmission, and have a greater chance of evolving to overt infection. MRSA can colonize the skin and nostrils of hospital patients, healthcare professionals, and healthy individuals, as shown by a study in which 758 patients admitted to the Brooke Army Medical Center were swabbed for MRSA on admission.^29^ In this study, 3.4 % of the swabs were positive for MRSA, a 13-fold increase in the risk of infection in comparison with patients who were not colonized on admission (19 % of the colonized patients developed an infection).

A meta-analysis and cohort study found that patients infected by MRSA suffered higher mortality when they presented bacteremia, even though 77.4 % of the published studies were unable to demonstrate conclusively the impact of MRSA on mortality.^30^ Once the studies were combined for analysis, however, the association between methicillin resistance and mortality in patients with *S. aureus* bacteremia became clear, with longer length of hospitalization and higher healthcare costs in comparison with patients infected with MSSA. In the present study, the 30-day mortality rate in the MRSA sample (30 %) was higher than that recorded in the MSSA group (20 %), but the difference was not significant, although this may have been a consequence of the small sample size. There was a similar proportion of MRSA and MSSA infections in patients who had severe complications, such as ICU admission, need for mechanical or non-invasive ventilation, the use of amines and acute renal failure.

### Study limitations

One of the major limitations of the present study was its retrospective nature, with incomplete data or no information particularly regarding sociodemographic parameters. The small sample size in a single center which deals mainly with the treatment of patients with HIV/AIDS are also potential limitations. Data from one infectious disease unit in a specific region of Brazil limits generalizability to other hospitals, geographic regions, or healthcare systems. Despite these limitations, the findings presented here provide important insights into different aspects of serious staphylococcal infection, especially regarding PLHIV, a group for which few published data are available on staphylococcal infections.

## Conclusions

The most common comorbidity of serious staphylococcal infections identified in the present study was HIV/AIDS. Patients with *S. aureus* and HIV infections were much younger than those in non-HIV patients with *S. aureus* infection and the mean CD4 cell count was 95 cells/mm^3^, consistent with the reported threshold of 200 cells/mm^3^ as a risk factor for staphylococcal infections. However, no significant differences were found in serious outcomes comparing PLHIV and non-HIV patients, although all the cases of relapse and reinfection were recorded in the PLHIV group. Secondly, the main portal of entry for *S. aureus* bacteremia were central venous catheters, while chronic dermatological conditions were the main portal for the other infection sites. Overall, 53.7 % of the infections were community-acquired, while the other 46.2 % were acquired in hospital. One in five PLHIV were colonized by MRSA on admission. MRSA infections accounted for around a third of cases whilst MSSA infections were present in over two-thirds of cases. All MRSA isolates were susceptible to cotrimoxazole, and were, therefore, ca-MRSA phenotypically. Almost half of the nosocomial *S. aureus* isolates had the ca-MRSA pattern based on their susceptibility to cotrimoxazole. These data are important regarding transition to oral antibiotics when treating these infections. The 30-day mortality in the MRSA infections was higher than in the MSSA group but this was not statistically significant.

## Funding

This study is a result of a Master's Degree by Dr. Narendra Valobdas in the Postgraduate course in Infectious Diseases at Instituto Nacional de Infectologia Evandro Chagas, Fiocruz, Rio de Janeiro, Brazil, who provided funding for this work.

## Conflicts of interest

The authors declare no conflicts of interest.

## Appendix Supplementary materials

Image, application 1
