# Supplementary material for: Severe Staphylococcus aureus infection: associated factors and outcomes
Source: Braz J Infect Dis. 2025 Aug 9;29(5):104573. doi: 10.1016/j.bjid.2025.104573 (PMC12357306; doi:10.1016/j.bjid.2025.104573)
Supplement: Supplementary file 1 [file mmc1.docx]

**BJID-D-25-00023**

**Supplementary Material**

| **1. DATA COLLECTION FORM** | | | | | |
| --- | --- | --- | --- | --- | --- |
| **Collection of data on the colonization and infection profile of staphylococcus aureus in HIV/AIDS patients in the infirmary of the Instituto Nacional de Infectologia Evandro Chagas, Rio de Janeiro, between 2016 and 2021** | | | Form no.:_______  Initials: ________ | | |
| Inclusion criteria: adult patients (≥18-years-old) admitted to hospital with infection or colonization by S. aureus (exclusion criteria: <18-years-old, palliative care, pregnancy, crianças). | | | | | |
| Date of internment: ........../............/.......................... | | | | | |
| Date of infection: ........../............/............................ | | | | | |
|  | | | | | |
| **RECENT HISTORY OF THE PATIENT:** | | | | | |
| Transferred from another hospital ( ) no/( ) yes, name of the hospital of origin: ................. | | | | | |
| Admitted to hospital within the past 3-months ( ) no; ( ) yes | | | | | |
| Number of days of hospitalization in the preceding hospital: | | | | | |
| Hemodialysis? ( ) yes ( ) no | | | | | |
| Resident in long-term institution: ( ) no; ( ) yes | | | | | |
|  | | | | | |
| **B. SOCIODEMOGRAPHIC PROFILE:** | | | | | |
| Date of birth ..../..../........ No information ( ) | | | | | |
| Sex: ( ) female = 0 ( ) male = 1 | | | | | |
| Ethnicity: ....................................................................................... No information ( ) | | | | | |
| White ( ); black ( ); brown ( ); yellow ( ); indigenous ( ) (see SISCEL or other official identification document of the patient) | | | | | |
| Birthplace: ........................................................................................no information ( ) | | | | | |
| Residence (neighborhood): | | | | | |
| Lives in subnormal clusters: | | | | | |
| Marital status: ..................................................................................no information ( ) | | | | | |
| Occupation/profession (CBO: Brazilian Occupation Code): | | | | | |
| Healthcare worker: yes ( ); no ( ); no information ( ). | | | | | |
| Informal work ( ); formal employment ( ); unemployed ( ); retired ( ) | | | | | |
| Education: primary school 1 ( ); primary school 2 ( ); high school ( ); college graduate ( ); illiterate ( ) | | | | | |
| Family income: less than 1 minimum wage ( ); 1–3 minimum wages; ( ); more than 3 minimum wages ( ); Welfare ( ). | | | | | |
| Number of rooms in residence: | | | | | |
| Number of people that live in the same residence: | | | | | |
| Lives with a healthcare worker ( ) no; yes ( ). | | | | | |
|  | | | | | |
| **C. LIFESTYLE:** | | | | | |
| Smoker: yes ( ); no ( ); no information ( ). | | | | | |
| Tobacco load (packs/year): .......................................................................................... | | | | | |
| Drinker: yes ( ); no ( ); no information ( ). | | | | | |
| How many units of alcohol per week/drinking since when:........................................... | | | | | |
| Use of other types of drugs? No ( ); no information ( ) yes ( ), which one(s): ( ) crack; ( ) cocaine; ( ) inhaled; ( ) cannabis; ( ) other injectable drug | | | | | |
| Daily or weekly use? ( ) yes ( ) no | | | | | |
|  | | | | | |
| **D. COMORBIDITIES:** (*Ceclin – Servlab – Immunodiagnosis) | | | | | |
| Chronic dermatopathy (for example: eczema, psoriasis): | | | | | |
| Current hepatitis C: yes ( ); no ( ); no information ( ) | | | | | |
| Chronic hepatitis B (positive HBsAg): yes ( ); no ( ); no information ( ) | | | | | |
| HTLV: yes ( ); no ( ); no information ( ) | | | | | |
| Diabetes mellitus: no ( ); no information ( ), yes ( ), medication:.................................... | | | | | |
| Recent cancer (<1 year): no ( ); no information ( ); yes ( ) | | | | | |
| *if yes, describe the location of the cancer treatment given: | | | | | |
| Chemotherapy | | | | | |
| Radiotherapy | | | | | |
| Steroids | | | | | |
| Use of steroids or chemotherapy during the infection: | | | | | |
| No ( ); ( ) yes, no information ( ); which type: .................................................... | | | | | |
| Chronic renal failure: yes ( ); no ( ); no information ( ) | | | | | |
| Chronic hepatopathy: yes ( ); no ( ); no information ( ) | | | | | |
| Congestive Heart Failure | | | | | |
| Coronary Artery Disease (CAD): yes ( ); no ( ); no information ( ) | | | | | |
| Systemic arterial hypertension: yes ( ); no ( ); no information ( ) | | | | | |
| Chronic Obstructive Pulmonary Disease: yes ( ); no ( ); no information ( ) | | | | | |
| Autoimmune disease: yes ( ); no ( ); no information ( ) | | | | | |
| Transplant: yes ( ); no ( ); no information ( ) | | | | | |
| Other comorbidities:...................................................................................... | | | | | |
|  | | | | | |
| **E. HISTORY OF INFECTION BY HIV:** | | | | | |
| HIV: yes ( ); no ( ); no information ( ) | | | | | |
| *new case of HIV no ( ); no information; yes ( ); date of the diagnosis: ............ | | | | | |
| (SISCEL, CECLIN, patient filter, verify the evolution of the ambulatorial consultations in infectology) | | | | | |
| CD4 –nadir CD4 count: ............................. .... / no ( ) / no information ( ) | | | | | |
| *CECLIM – SERVLAB – draft—no. of the medical record – multi-user. | | | | | |
| Most recent CD4 count since hospitalization/date: .............. / no ( ) / no information ( ) | | | | | |
| Most recent CD8/date: ........................................................ / no ( ) / no information ( ) | | | | | |
| CD4:CD8 ratio (divide CD4 by CD8): .......................................... | | | | | |
| Most recent viral load: ................................................. / no ( ) / no information ( ) | | | | | |
| Date of the results:…/..../......... | | | | | |
| Use of ART (verify this information on the internment form and/or CECLIN-filter of patient progress in the infetious diseases outpatient unit): | | | | | |
| yes ( ); no ( ); no information ( ). | | | | | |
|  | | | | | |
| **USE OF PRIMARY AND/OR SECONDARY PROPHYLAXIS FOR OPPORTUNISTIC INFECTIONS:** | | | | | |
| Cotrimoxazole: yes ( ); no ( ); no information ( ) | | | | | |
| Azithromycin: yes ( ); no ( ); no information ( ) | | | | | |
| *If yes, which medications: (verify this information on the internment form and/or CECLIN-filter of patient progression in the ambulatory for infectology, ambulatorial record). | | | | | |
|  | | | | | |
| **OPPORTUNISTIC DISEASES UNDERGOING TREATMENT AT THE TIME OF THE INFECTION:** | | | | | |
| Tuberculosis: yes ( ); no ( ); no information ( ) | | | | | |
| Toxoplasmosis: yes ( ); no ( ); no information ( ) | | | | | |
| Cryptococcosis: yes ( ); no ( ); no information ( ) | | | | | |
| Sporotrichosis: yes ( ); no ( ); no information ( ) | | | | | |
| Cytomegalovirus: yes ( ); no ( ); no information ( ) | | | | | |
| Esophageal candidiasis: yes ( ); no ( ); no information ( ) | | | | | |
| Presence of prurigo: yes ( ); no ( ); no information ( ). | | | | | |
|  | | | | | |
| **F. PROFILE OF COLONIZATION AND INFECTION BY STAPHYLOCOCCUS AUREUS** | | | | | |
| Presence of colonization by Staphylococcus aureus at the time of hospital admission: ( ) no; ( ) no information; ( ) yes | | | | | |
| Presence of infection by Staphylococcus aureus at the time of hospital admission: ( ) no; ( ) no information; ( ) yes | | | | | |
|  | | | | | |
| **Portals of entry: (7 DAYS)** | | | | | |
| Chronic dermatological condition (for example: eczema, psoriasis, prurigo): | | | | | |
| Lesions caused by insect bites: yes ( ); no ( ); no information ( ) | | | | | |
| Trauma: yes ( ); no ( ); no information ( ) | | | | | |
| Tattoos: yes ( ); no ( ); no information ( ). | | | | | |
| Piercing: yes ( ); no ( ); no information ( ). | | | | | |
| Burns: yes ( ); no ( ); | | | | | |
| Peripheral vascular catheter: yes ( ); no ( ); no information ( ) | | | | | |
| Surgery: yes ( ); no ( ); no information ( ) | | | | | |
| Short-term deep vascular catheter: yes ( ); no ( ); no information ( ) | | | | | |
| Vascular catheter for hemodialysis: yes ( ); no ( ); no information ( ) | | | | | |
| Long-term vascular catheter: yes ( ); no ( ); no information ( ) | | | | | |
| Pacemaker: yes ( ); no ( ); no information ( ) | | | | | |
| Valvular prosthesis: yes ( ); no ( ); no information ( ) | | | | | |
| Orthopedic prosthesis: yes ( ); no ( ); no information ( ) | | | | | |
| Time of use of invasive device: .................................. | | | | | |
| Other: yes ( ); no ( ); | | | | | |
| Unknown portal of entry: yes ( ); no ( ). | | | | | |
|  | | | | | |
| **INFECTION SITE (STAPHYLOCOCCUS AUREUS)**: (indicate all the categories that apply) | | | | | |
| Skin and soft tissue infection ( ); muscle ( ); | | | | | |
| Bacteremia ( ); endocarditis ( ); visceral abscesses ( ); meningoencephalitis ( ); osteomyelitis ( ); septic arthritis ( ); endophthalmitis ( ) | | | | | |
| Other forms ( ); which:................................ | | | | | |
|  | | | | | |
| **STAPHYLOCOCCUS AUREUS ANTIBIOTIC SUSCEPTIBILITY PROFILE** (fill in susceptible or resistant, and when resistant to vancomycin or daptomycin, include the MIC): MRSA ( ) or MSSA ( ) | | | | | |
| Oxacillin ( ); vancomycin ( ) MIC _____; linezolid ( ); daptomycin ( ) MIC ___; cotrimoxazole ( ); doxycycline ( ); clindamycin ( ). | | | | | |
| Allergy to any antibiotic: yes ( ); no ( ); no information ( ) | | | | | |
| If yes, which one? Penicillin ( ); cephalosporin ( ); sulphas ( ) | | | | | |
| Main intravenous antibiotics used to treat S. aureus: | | | | | |
| Oxacillin ( ); cefazolin ( ); vancomycin ( ); teicoplanin ( ); daptomycin ( ); cotrimoxazole ( ); clindamycin ( ); linezolid ( ). | | | | | |
| Number of days of treatment: ........ Date of the start and end of the treatment | | | | | |
| Main oral antibiotics used to treat S. aureus: | | | | | |
| Amoxicillin/clavulanate ( ); cefuroxime ( ); cotrimoxazole ( ); clindamycin ( ); linezolid ( ); doxycycline ( ). | | | | | |
| Total treatment time: Date of the start and end of treatment | | | | | |
| If the focus of the treatment was an abscess, was it drained? yes ( ); no ( ); no information ( ). | | | | | |
| Findings relevant to the diagnosis: | | | | | |
| Echocardiogram: yes ( ); no ( ); no information ( ) | | | | | |
| Findings compatible with endocarditis (Vegetations/new valvular regurgitation/cardiac abscess): yes ( ); no ( ); no information ( ) | | | | | |
| Other radiological images: yes ( ); no ( ); no information ( ) | | | | | |
| which ones: | | | | | |
| Simple X-Ray: yes ( ); no ( ); no information ( ). | | | | | |
| Ultrasonography: yes ( ); no ( ); no information ( ). TC: yes ( ); no ( ); no information ( ), | | | | | |
| Magnetic Resonance Imaging (MRI): yes ( ); no ( ); no information ( ). | | | | | |
| Findings compatible with osteomyelitis: yes ( ); no ( ); no information ( ). | | | | | |
| Pyomyositis: yes ( ); no ( ); no information ( ). | | | | | |
| Findings compatible with staphylococcal infection in fundoscopy: yes ( ); no ( ); no information ( ). | | | | | |
| Decolonization during hospitalization yes ( ); no ( ); no information ( ). | | | | | |
|  | | | | | |
| **Outcome:** | | | | | |
| ICU admission: yes ( ); no ( ); no information ( ). | | | | | |
| Amines: yes ( ); no ( ); no information ( ). | | | | | |
| Hemodialysis: yes ( ); no ( ); no information ( ). | | | | | |
| Mechanical ventilation: yes ( ); no ( ); no information ( ). | | | | | |
| Non-invasive ventilation: yes ( ); no ( ); no information ( ). | | | | | |
| Date of release from hospital (..../..../........) | | | | | |
| Dates of ICU admission and discharge (..../..../........) - (..../..../........) | | | | | |
| Length of ICU admission (in days): | | | | | |
| Death in hospital: yes ( ); no ( ); Date of death: (..../..../........) | | | | | |
| Death in up to 30 days: yes ( ); no ( ); No information ( ). | | | | | |
|  | | | | | |
| **H. PHYSICAL EXAMS:** | | | | | |
| *(Admission – nutrition – find data such as height and weight). | | | | | |
| Weight (nearest to the time of diagnosis of the infection by S. aureus): .................. | | | | | |
| Height: ................... No information ( ) | | | | | |
| Body Mass Index (BMI): (kg/m^2^) .......................................... No information ( ). | | | | | |
| Attention!! Try to provide all these data. | | | | | |
| Vital signs at the time of hospital admission: | | | | | |
| *(CECLIN-patient filter-draft-ambulatorial progression-see progression in the infirmary on the date of internment) | | | | | |
| Temperature: | | Systolic pressure: | | | |
| Respiratory frequency: | | Diastolic pressure: | | | |
| Heart rate: | | Mean Arterial Pressure: (Calculate with the app): | | | |
| Complete with MEWS and qSOFA | | | | | |
| Laboratory exams – up to 72 hours from the time of the infection, and if blood culture (BC) positive, on the day the BC was taken. | | | | Form no.:  Initials: | |
| D7=7 days of effective treatment. | | | | | |
| Date: (.... /.... /......) | Exams | | | | No information |
|  | Hemoglobin: | | | |  |
|  | Leukocytes: | | | |  |
|  | Neutrophils: (segmented and bands). | | | |  |
|  | Absolute value: percentage % / Bands (%) | | | |  |
|  | Platelets: | | | |  |
|  | C-Reactive Protein: | | | |  |
|  | Lactate: | | | |  |
|  | Creatinine: | | | |  |
|  | Urea: | | | |  |
|  | Creatinine Clearance: | | | |  |
|  | Total bilirubin: | | | |  |
|  | Albumine | | | |  |
|  | Glycemia | | | |  |
|  | Glycated hemoglobin (at any time during the internment) | | | |  |
| Ceclin – Servlab – draft – number of medical record – old data – include the desired exam | | | | | |
| Relapse of infection in up to 30 days, new infection, in 90 days. | | | | | |
|  | | | | | |
| Completed by and date: | | | | | |
| ......................................................................................................... (..../..../........) | | | | | |

**Supplementary Table 1** Clinical outcomes in patients with Staphylococcus aureus infection stratified by 30-day mortality and survival.

| **Clinical parameter** | **Number^a^ (%) of patients in group** | | **p** |
| --- | --- | --- | --- |
|  | **Death in 30 days (n=15)** | **Survived (n=52)** |  |
| ICU admission (27) | 12 (80.0%) | 15 (28.8%) | 0.001 |
| Use of amines (vasopressors) | 12 (80.0%) | 7 (13.4%) | <0.001 |
| Mechanical ventilation | 12 (80.0%) | 7 (13.4%) | <0.001 |
| Non-invasive ventilation | 5 (33.3%) | 7 (13.4%) | 0.046 |
| Acute kidney failure in ICU (hemodialysis) | 6 (40.0%) | 11 (21.1%) | 0.207 |
| Median (IQR) time in ICU (days) | 13 (8–22.25) | 11 (4–17) | 0.231 |

^a^ Except for time in ICU, which was measured in days, with the (IQR).

ICU, Intensive Care Unit; IQR, Interquartile Range.

**Supplementary** **Table 2** Comorbidities of patients with S. aureus infection stratified by 30-day mortality and survival.

| **Variable** | **Number (%) of patients with condition in group** | | | **p** |
| --- | --- | --- | --- | --- |
|  | **Total (n=67)** | **Survived (n=52)** | **Death in 30 days (n=15)** |  |
| Recent cancer | 5 (7.6%) | 2 (3.9%) | 3 (20%) | 0.130 |
| Cancer treatment | 7 (10.6%) | 5 (9.8%) | 2 (13.3%) | 1.000 |
| Chemotherapy | 3 (4.5%) | 2 (3.9%) | 1 (6.7%) | 1.000 |
| Radiotherapy | 4 (6.2%) | 3 (6%) | 1 (6.7%) | 1.000 |
| Use of corticoids | 5 (7.6%) | 4 (7.8%) | 1 (6.7%) | 0.889 |
| HTLV | 6 (9.1%) | 4 (7.8%) | 2 (13.3%) | 0.863 |
| Diabetes mellitus | 12 (18.2%) | 10 (19.6%) | 2 (13.3%) | 0.686 |
| Congestive Cardiac Failure | 5 (7.6%) | 3 (5.9%) | 2 (13.3%) | 1.000 |
| Systemic Arterial Hypertension | 9 (13.6%) | 7 (13.7%) | 2 (13.3%) | 0.512 |
| Chronic Obstructive Pulmonary Disease | 1 (1.5%) | 0 (0%) | 1 (6.7%) | 1.000 |
| Coronary Arterial Disease | 1 (1.5%) | 1 (2%) | 0 (0%) | 0.378 |
| Chronic dermatopathy^a^ | 6 (9.1%) | 6 (11.8%) | 0 (0%) | 1.000 |
| Hepatitis C | 1 (1.5%) | 1 (2%) | 0 (0%) | 0.235 |
| Chronic Renal Disease | 8 (12.1%) | 8 (15.7%) | 0 (0%) | 1.000 |
| Chronic hepatopathy | 2 (3.0%) | 2 (3.9%) | 0 (0%) | 0.238 |
| Other comorbidities^b^ | 19 (28.8%) | 17 (33.3%) | 2 (13.3%) |  |

^a^ Chronic dermatopathy was defined as any case in which a patient presented a cutaneous lesion continuously for more than six weeks, and was present at the time of S. aureus infection or hospitalization (for example, patients with psoriasis, chronic dermatitis, eczema, prurigo, and granulomatous skin diseases).

^b^ Other comorbidities included alopecia, glaucoma, transversal myelitis, Guillain-Barré syndrome, hernia, and gastritis.

**Supplementary Table 3** Physical and laboratory data of patients with Staphylococcus aureus infection up to 72-hours of admission.

|  | **Mean (Interquartile Range) values recorded for group** | | |  |
| --- | --- | --- | --- | --- |
| **Parameter** | **Total (n = 67)** | **Death in 30 days (n = 15)** | **Survived (n = 52)** | **p** |
| Weight (kg) | 60 (53–72) | 58.0 (52.75‒64.1) | 60.05 (53.30‒76.90) | 0.315 |
| Height (m) | 1.68 (1.58–1.70) | 1.60 (1.50–1.70) | 1.69 (1.60–1.70) | 0.053 |
| Body Mass Index (BMI) | 22.5 (21.0–26.4) | 22.6 (20.0–26.0) | 22.5 (21.7–25.3) | 0.773 |
| Temperature (°C) | 36.9 (36.0–38.0) | 36.5 (36.0–37.3) | 37.1 (36.2–38.1) | 0.067 |
| Mean arterial pressure (mmHg) | 83.33 (73.30–96.50) | 86.00 (73.16–91.83) | 83.33 (74.20–96.70) | 0.510 |
| Respiratory frequency (breaths per minute) | 22 (20–25) | 24.0 (18.5–27.5) | 22 (20–24) | 0.484 |
| Heart rate (Beats per minute) | 98.0 (89.3–109.8) | 104.0 (97.5–113.5) | 95.0 (86.5–107.0) | 0.101 |
| Hemoglobin (g/dl) | 9.5 (8.1–11.2) | 9.7 (8.4–10.8) | 9.1 (8.0–11.4) | 0.963 |
| Leukocytes (mm^3^) | 9,470 (6,480–14,397) | 8,610 (6,705–15,615) | 9,520 (6,210–14,345) | 0.771 |
| Segmented neutrophils (absolute values ‒ mm^3^) | 6,759 (3,927–9,774) | 7,253 (5,756.5–10,810) | 7,253 (5,054–10,810) | 0.523 |
| Band forms, absolute (mm^3^) | 305 (128–1,011) | 396 (104–1,300) | 304 (165–713) | 0.963 |
| Platelets (mm^3^) | 222,500 (160,250–358,000) | 189,000 (98,500–259,500) | 252,000 (171,000–364,500) | 0.178 |
| C-Reactive Protein (mg/L) | 13.0 (7.4–22.0) | 10.84 (7.2–19.9) | 14.1 (7.6–22.0) | 0.592 |
| Creatinine (mg/dL) | 1.12 (0.8–2.2) | 1.09 (0.8–1.9) | 1.22 (0.9–2.5) | 0.488 |
| Urea (mg/dL) | 50.5 (27.5–81.0) | 76.0 (35.0–106.8) | 47 (24–68) | 0.098 |
| Total bilirubin (mg/dL) | 0.63 (0.3–1.6) | 0.31 (0.2–1.6) | 0.73 (0.4–1.5) | 0.267 |
| Glycemia (mg/dL) | 105 (96–139.5) | 106 (90–144) | 105 (96–134) | 0.985 |
